# Molecular Interplay of Small Molecules and Calcium Ions with α‑Synuclein Revealed by NMR and Molecular Dynamics Simulations

**DOI:** 10.1021/acschemneuro.6c00106

**Published:** 2026-03-24

**Authors:** Filippo Turchi, Haydar Taylan Turan, Marco Schiavina, Giuseppe Brancato, Isabella C. Felli, Roberta Pierattelli

**Affiliations:** ∇ Department of Chemistry “Ugo Schiff” and Magnetic Resonance Center (CERM), 9300 University of Florence, Via L. Sacconi 6, 50019 Sesto Fiorentino, Italy; ‡ 19004 Scuola Normale Superiore, Piazza dei Cavalieri 7, 56126 Pisa, Italy

**Keywords:** Drug discovery, Synucleinopathies, Intrinsically Disordered Proteins, Protein interactions, Amino acids side chain, ^13^C NMR

## Abstract

Human α-synuclein is an intrinsically disordered protein concentrated at presynaptic terminals and strongly linked to Parkinson’s disease and other synucleinopathies. Its dynamic C-terminal region mediates interactions with small molecules and metal ions. Here, we used high-resolution nuclear magnetic resonance spectroscopy (NMR) and molecular dynamics (MD) simulations to characterize interactions between the C-terminal α-synuclein construct, the small molecule fasudil, and calcium ions. NMR data show that fasudil and Ca^2+^ bind preferentially to overlapping regions enriched in alternating tyrosine and acidic residues while preserving the protein’s disordered nature. Side-chain-resolved spectra indicate distinct driving forces for fasudil and calcium binding. MD simulations reveal that Ca^2+^ modifies the local electrostatic environment, decreasing fasudil interaction frequency through electrostatic screening and steric effects. Despite this, fasudil retains dynamic, reversible contacts with key tyrosine residues. Overall, exposed α-synuclein conformations allow simultaneous, ligand-specific interactions, highlighting side-chain hotspots governing binding in Ca^2+^-rich conditions.

## Graphical abstract

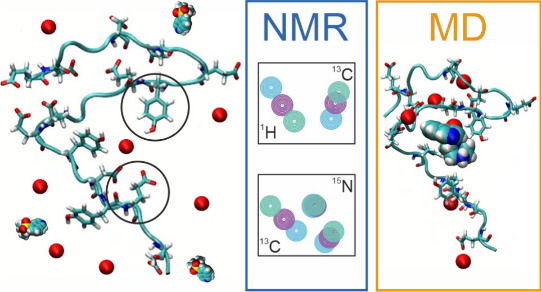

Intrinsically disordered proteins (IDPs) are a large class of proteins characterized by the lack of a three-dimensional structure and are extensively dynamic in their native state. Despite the absence of a stable fold, these highly flexible proteins play relevant roles in a variety of different cellular pathways. IDPs are also often linked to the onset of incurable diseases, such as neurodegenerative ones.^1^

Human α-synuclein (α-syn) is an IDP located predominantly at the presynaptic terminals.^2^ It is involved in several neurodegenerative disorders collectively known as synucleinopathies also including Parkinson’s disease (PD).^3–8^ It is constituted by 140 amino acids, divided into three main regions: the N terminus (1–60), rich in positively charged amino acids, a more hydrophobic central region (61-94) known as NAC (non-amyloid-β component) and the C-terminal tail (95–140) characterized by the presence of several negatively charged residues (15 aspartate/glutamate residues out of 45).^6,9–11^ The protein, particularly the latter region, is targeted by small molecules, whose interactions have been investigated as part of ongoing efforts to develop pharmacological strategies aimed at preventing or reversing the formation of protein aggregates and fibrils, known to play a role in the pathogenesis of PD.^12–15^ A large body of evidence suggests that these compounds do not induce their target to adopt well-defined conformations upon binding; rather, α-syn remains largely disordered during its interaction with the ligands.^16^ A dynamic network of transient interactions, which results in minimal perturbations of the conformational ensemble of α-syn, are thus responsible for ligand affinity, as recently proposed.^17,18^ Consequently, it may not be feasible to identify only a small number of representative ligand-binding modes to serve as starting points for traditional structure-based drug design approaches.

Among the potential drug compounds, the small molecule fasudil [5-(1,4-diazepane-1-sulfonyl) isoquinoline] was found to be neuroprotective in mouse models of PD and to interact with monomeric α-syn in the mM range *in vitro*, by various techniques, including nuclear magnetic resonance (NMR) spectroscopy.^12,18,19^ The latter has clearly demonstrated, through the observation of chemical shift perturbations (CSPs), that fasudil interacts preferentially to α-syn’s C-terminal tyrosine residues Y133 and Y136, thereby reducing its aggregation propensity.^12^ This dynamic binding, supported by site directed mutagenesis and computational studies, reveals fasudil as a promising modulator of α-syn pathology.^12,18^

In addition to small molecules like fasudil, various divalent cations are also known to interact with α-syn.^20–26^ In particular, calcium ions have been shown in multiple studies to bind to the C-terminal region of α-syn, which is rich in negatively charged residues such as aspartate and glutamate.^24,26–28^ NMR experiments have emphasized the role of these acidic side chains in mediating calcium binding and suggest the presence of simple structural motifs that may facilitate this interaction.^24^ Under physiological conditions, transient calcium binding is thought to contribute to the regulation of α-syn’s normal synaptic function, particularly in modulating neurotransmitter release through its interaction with synaptic vesicles.^23^ The calcium ions fluctuations regulating the interaction with α-syn can reach up to several hundreds of μM in healthy neurons upon neuronal stimulation as a result of a concomitant calcium influx via voltage-gated calcium channels.^23^ Moreover, dysregulated calcium levels can trigger toxic aggregation pathways, promoting the progression of neurodegeneration.^27^

Therefore, investigating the concurrent interactions of α-syn with both calcium ions and fasudil is relevant for elucidating the molecular properties of this protein and may uncover novel therapeutic strategies for targeting synucleinopathies. High-resolution techniques, including ^1^H and ^13^C NMR spectroscopy and molecular dynamics (MD) simulations, are here employed to characterize the interaction of α-syn with fasudil in the presence of calcium ions at the atomic level, with particular attention to the role of the side chains of the involved amino acids. Indeed, NMR experiments that focus on the extreme edge of amino acid side chains are crucial to dissect the driving forces guiding the interactions with different partners (i.e., fasudil and Ca^2+^) providing additional information to that available from experiments monitoring backbone resonances only.

Since both calcium ions and fasudil interact with the C-terminal region of the protein,^12,18,23–25^ a construct comprising residues 112–140 of α-syn (C-α-syn) is used as a model for the full-length protein in a combined experimental and computational study. While transient interdomain interactions in the full-length protein could, in principle, influence ligand binding, the data on Ca-binding (Figure S1 and Figure S2) indicate that C-α-syn successfully recapitulates the binding properties of the full-length protein. A more extended C-terminal fragment (e.g., the canonical residues 96–140) is not expected to provide additional information regarding our specific targets, while it may complicate the isolation and analysis of the specific interactions occurring in the 112–140 region. Our construct therefore serves as a valid model to analyze binding events that are localized within the C-terminal region of α-syn while minimizing spectral complexity. This choice enables also more efficient MD simulations, providing improved statistics for the populations of intermolecular interactions.

Figure 1 reports the CSP occurring to the ^1^H and ^15^N backbone signals of selected residues of C-α-syn upon the addition of calcium ions. As illustrated in Figure 1A, 1B for a subset of peaks (full spectra are shown in Figure S3A), the largest chemical shift changes occur in the backbone signals of A124, Y125, E126, M127, S129, Y136, and E137 similarly to the wild-type protein, with comparable changes in both the identity of the affected residues and the extent of the perturbations observed (Figure S1).

**Figure 1:**
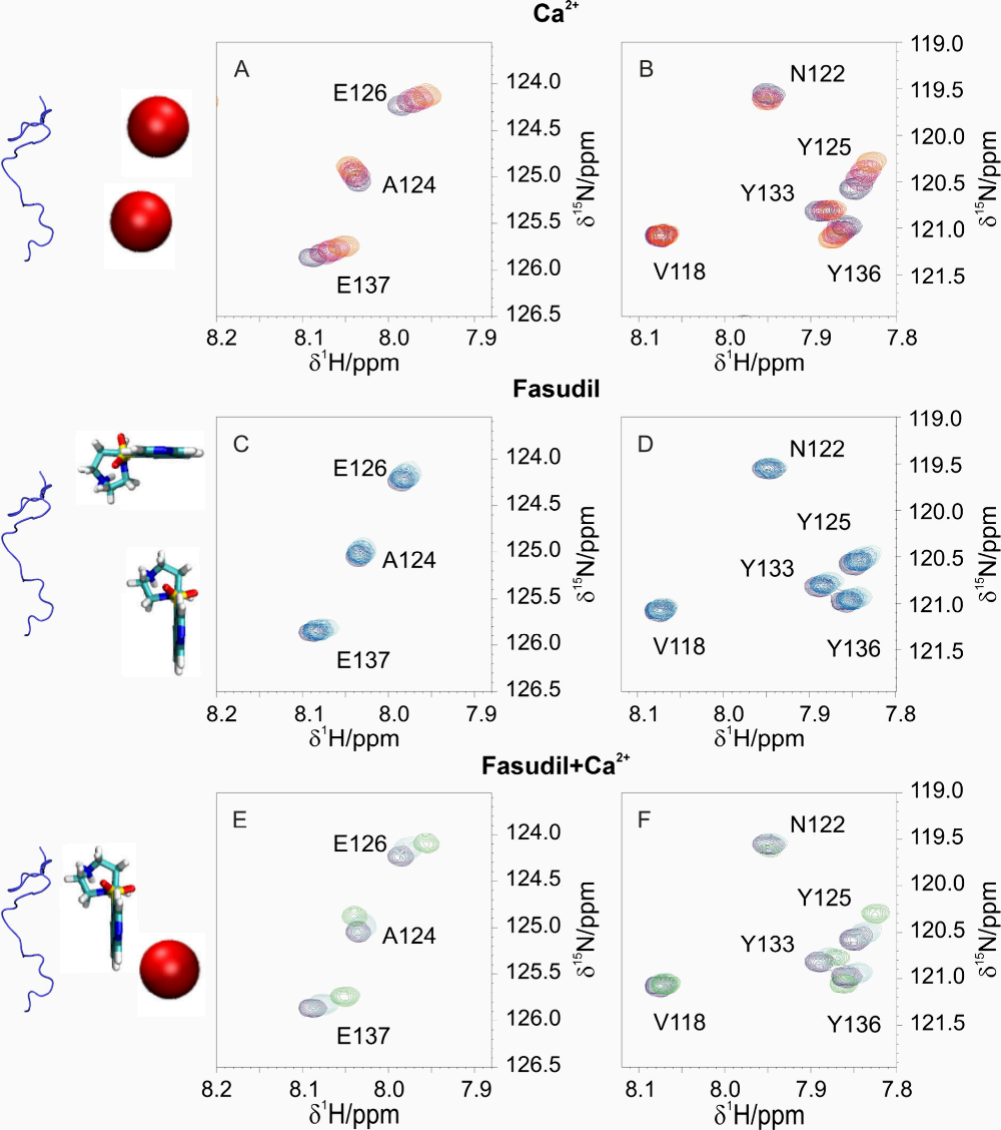
2D HN NMR spectra obtained upon the addition of calcium ions (panels A and B), fasudil (panels C and D) and both fasudil and Ca^2+^ (panels E and F) to a solution containing 0.2 mM of ^15^N C-α-syn. Panels A and B show in dark blue, violet, red, and orange the reference spectrum and the spectra of C-α-syn in the presence of 0.8, 1.6, and 3.2 mM Ca^2+^, respectively. Panels C and D show in dark blue, blue, light blue, and pale blue the reference spectrum and the spectra of C-α-syn in the presence of 0.8, 1.6, and 3.2 mM of fasudil, respectively. Panels E and F show, in dark blue, pale blue, and light green, the reference spectrum and the spectra with 3.2 mM fasudil and 3.2 mM fasudil/3.2 mM Ca^2+^, respectively. The latter set of spectra shows distinct chemical shift perturbations, a clear indication of the concurrent protein interaction with both ligands when present in solution.

The addition of fasudil to a C-α-syn solution induces only minor changes in the spectra, consistent with previous reports on the full-length protein.^12^ The most perturbed signals correspond to residues Y125, E126, M127, S129, Y133, Q134, and Y136, highlighting the involvement of a very similar region to that involved in sensing calcium ions (Figure 1C, 1D and S3B).

Notably, both tyrosine residues and negatively charged residues in the C-terminal region are perturbed in the presence of either ligand, as observed through changes in backbone amide nitrogen and proton resonances (Figure 1A-D). Not surprisingly, the same resonances are affected by the simultaneous presence of both ligands (Figure 1E, 1F and S3C). Comparable trends are evident in the 2D CACO spectra, which enable to monitor the other two nuclei of the backbone ^13^C^α^ and ^13^C′. The chemical shift variations for all the detected nuclei are reported in Figure S4. The final spectra of the sample containing the three components (C-α-syn, Ca^2+^ and fasudil) are superimposable, regardless of the order in which calcium ions and fasudil are added to the solution, confirming the same thermodynamic chemical equilibrium is reached (Figure S5).

Side chains are generally the primary players in interactions; monitoring the chemical shifts of nuclei constituting amino acid side chains thus provides direct access to the main interaction site, a feature that is particularly relevant for highly solvent-exposed IDPs. To this end, the 2D CACO experiment, which yields information on side chains containing carboxylate or carbonyl groups (Asp and Asn residues through ^13^C^ß^-^13^C^γ^ resonances, Glu and Gln through ^13^C^γ^ -^13^C^δ^ resonances),^24,29^ enables mapping of the negatively charged carboxylate groups of Asp and Glu, which are the most relevant for the interaction with calcium ions.^24^

Given the importance of the aromatic residues, particularly the tyrosine residues (i.e., Y125, Y133, and Y136), we employed a modified 2D HC TROSY experiment tailored for aromatic side chains to obtain well resolved ^1^H^ε^-^13^C^ε^ and ^1^H^δ^-^13^C^δ^ resonances of tyrosine residues.^30^

Upon addition of calcium ions to a solution containing C-α-syn, the most pronounced effects are observed for peaks corresponding to acidic residues. This is particularly evident when focusing on the side chains of these negatively charged residues, as shown in Figure 2A. In contrast, aromatic residues exhibit only minor perturbations, with small chemical shift changes detectable in the 2D HC TROSY spectra (Figure 2B). On the other hand, in the presence of fasudil the 2D CACO spectra display only slight chemical shift changes (Figure 2C), while the 2D HC TROSY spectra reveal more substantial perturbations of the side chain resonances of tyrosine residues (Figure 2D).

**Figure 2:**
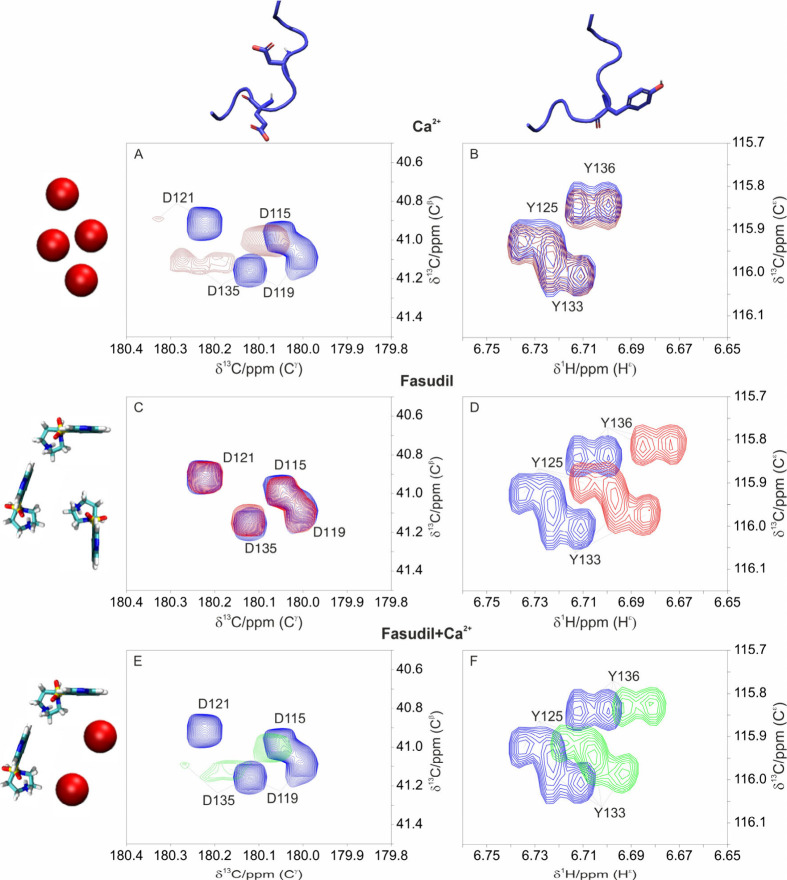
2D CACO NMR spectra, recorded on a 0.2 mM ^13^C–^15^N C-α-syn sample, highlighting the region where the resonances of aspartate side chains are found (panels A, C, and E) and 2D HC TROSY spectra of the region where the resonances of ^1^H^ε^–^13^C^ε^ are found (panels B, D, and F). Panels A–F show the superimposition of the spectra in the presence and absence of different compounds. The spectra always show in blue the free state of C-α-syn (0.2 mM protein). Panels A and B show the spectra of C-α-syn in a molar ratio of 1:16 protein to calcium ions (red, 0.2 mM protein and 3.2 mM Ca^2+^). Panels C and D show the spectra of C-α-syn in a molar ratio of 1:16 protein to fasudil (dark red, 0.2 mM protein and 3.2 mM fasudil). Panels E and F show the spectra upon addition of 16 equiv of fasudil and 16 equiv of calcium ions (green, 0.2 mM protein, 3.2 mM Ca^2+^ and 3.2 mM fasudil). The region corresponding to glutamate side-chain resonances in the 2D CACO NMR spectra is shown in Figure S6. The axes display the specific observed resonances, C^β^-C^γ^ for aspartate residues (panels A, C and E) and C^ε^-H^ε^ for tyrosine residues (panels B, D and F).

It is worth noting that when using only backbone resonances as observables, the same protein region was perturbed upon addition of calcium ions, fasudil, or both. This is likely due to a combination of direct effects and induced perturbations that modulate the backbone conformational ensemble. In contrast, access to direct information on interaction hotspots via side-chain resonances allows us to experimentally determine the roles of the different driving forces promoting the interactions, which are not easily detectable when focusing exclusively on the backbone: electrostatic interactions for calcium ions and aromatic interactions for fasudil. The highly solvent-exposed and flexible disordered region is thus able to interact simultaneously with two different ligands through the same portion of the primary sequence.

In a solution of C-α-syn containing both calcium ions and fasudil, the same shifts observed with each individual ligand are essentially retained indicating the presence of a ternary complex. In other words, chemical shift changes are observed for both tyrosine residues (mainly perturbed by addition of fasudil) as well as carboxylate groups (mainly perturbed by addition of calcium ions). In Figure 2F, the peak positions of the aromatic side chains partially revert toward those observed for the free C-α-syn form. The resulting 2D HC TROSY spectrum (Figure 2F) is indeed a 2D map similar to the one of C-α-syn in interaction with fasudil (Figure 2D) where an appreciable chemical shift perturbation of the free form can be observed even though this is slightly attenuated with respect to the fully bound form. The steric hindrance caused by small but significant modification of the side chain position of the neighboring negatively charged residues can in fact alter the accessibility of fasudil to the aromatic side chains of tyrosine residues.

Results from MD simulations closely mirror the experimental observations and provide deeper insights into the interplay between C-α-syn, fasudil and Ca^2+^. Several key findings emerged from the atomistic simulations. One notable feature of these interactions is their high degree of dynamics (see Figure 3). Unlike the classical lock-and-key model of drug–protein binding, calcium ions and/or fasudil exhibit a dynamic binding pattern, which is largely influenced by the intrinsically disordered nature of C-α-syn. Rather than binding to a single, well-defined site, each individual ligand can interact with multiple regions of the protein with varying frequencies throughout the simulation.

**Figure 3:**
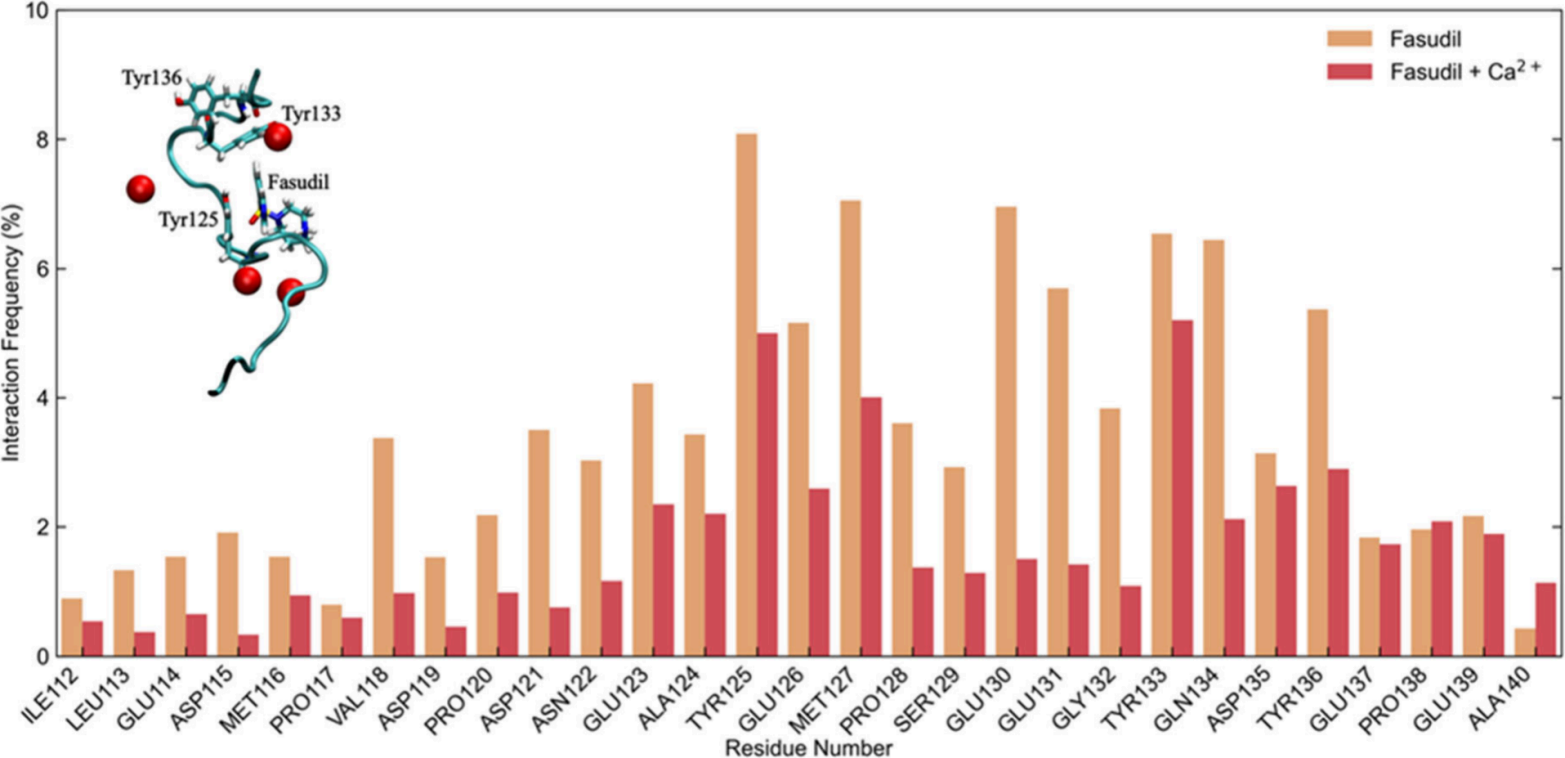
Interaction frequency (%) of fasudil with C-α-syn residues in the presence (red) or absence (orange) of Ca^2+^ ions (30 mM) as issuing from corresponding MD simulations. In the inset, a representative molecular structure of the fasudil-C-α-syn interaction in the presence of Ca^2+^ ions (red spheres). Addition of Ca^2+^ ions modulates the residue-specific binding pattern of fasudil across the whole protein segment.

Beyond van der Waals contacts and general hydrophobic interactions, several specific noncovalent interactions contribute significantly to fasudil’s binding profile (Figures S7 and S8), providing overall a rather heterogeneous ensemble of interactions. These include hydrogen bonds, π–π stacking and cation−π interactions with tyrosine residues, as well as electrostatic interactions with negatively charged Asp and Glu side chains. Among these, π–π stacking interactions between fasudil’s isoquinoline ring and tyrosine side chains (i.e., Y125, Y133, and Y136) are particularly relevant, displaying also a variety of binding modes (Figure S9). MD simulations also reveal multiple binding and unbinding events over time (see Figures S7 and S8), highlighting the transient and reversible nature of fasudil’s association with C-α-syn. This confirms that fasudil’s interaction with the protein is governed by a dynamic interplay of various short-lived interactions rather than long-lasting, stable binding.^18^ Interestingly, in addition to tyrosine residues, our computational analysis highlights recurrent and direct interactions of fasudil with M127 and Q134 and, to somewhat minor extent, with V118, N122, P128 and G132, in very good agreement with 2D NMR backbone and side-chain spectra (Figure 1 and Figures S3 and S6). In particular, M127 and Q134 can exert a cooperative effect with, respectively, Y125 and Y133 (see Figure S10), thus further stabilizing the protein–ligand interaction, mostly through hydrophobic contacts and hydrogen bonds (Figure S8). Indeed, 2D CACO NMR experiments allow to focus on the side-chain resonances (Figure S6A). Here Q134 (C^γ^-C^δ^ nuclei are observed) appears to be perturbed when fasudil is present in solution (Figure S6F), in line with MD simulations. A similar assisting role is played by a few acidic residues, e.g., E126 and D135, which are also located in the vicinity of tyrosine residues, although the nature of the interaction is basically electrostatic in this case.

Concerning the interaction between Ca^2+^ and C-α-syn, simulation results corroborated the view that glutamate and aspartate residues of the protein have the dominant role through relatively strong electrostatic forces (Figure S11). In particular, we observed a significant interaction probability with E137, E139, E130, E131, E123, and E126, among the glutamate residues (Figure S11A), and D119, D121, D135, among the aspartate residues (Figure S11B), thus matching well the NMR results. Moreover, in line with experimental observations, E114 and D115 exhibit relatively low interaction frequencies with Ca^2+^ compared to other glutamate and aspartate residues along the C-α-syn sequence, as shown in Figure S11. These trends were consistent across both Ca^2+^–residue and fasudil–residue interaction profiles, suggesting that this region is generally less favorable for binding despite being solvent exposed and featuring residues with binding properties.

MD simulations provided supporting evidence and additional insights into the modulation effect of Ca^2+^ toward protein-fasudil interaction. Calcium ions and fasudil molecules preferentially interact with distinct sites on the protein surface and can clearly interact simultaneously with C-α-syn even if both are present in solution. However, the presence of Ca^2+^ consistently led to a reduction of the interaction frequency between fasudil and C-α-syn, as illustrated in Figure 3. This attenuation can now be explained by the two previously mentioned interconnected mechanisms. First, the introduction of Ca^2+^ alters the local electrostatic landscape, particularly around regions rich in negatively charged residues, due to the strong ionic interactions between Ca^2+^ and side chains such as those of Asp and Glu. These changes can reduce stabilization effects for fasudil. A clear example is that in the absence of Ca^2+^, the protein–ligand interactions persist, on average, for about 2.8 ns, but this residence time decreases to approximately 2.1 ns when Ca^2+^ ions are present (Figure S12).

The same trend was also obtained by evaluating fasudil’s dissociation constants in the absence (K_d_ = 3.5 mM) and presence (K_d_ = 10.9 mM) of calcium ions. These results further support the idea that Ca^2+^ modifies the protein’s interaction landscape, reducing the stability of fasudil binding at key sites, while not abrogating the protein–ligand interaction. Second, Ca^2+^ ions contribute to increased steric hindrance, reducing fasudil’s access to key binding regions on the protein. This phenomenon is especially evident in the vicinity of Tyr residues that play a central role in fasudil’s binding (Figure 3). Y125, for instance, is neighbored by E123 and E126; Y133 by E131 and D135; and Y136 by D135 and E137. In turn, such a phenomenon is also mutually observed on protein-Ca^2+^ interaction upon addition of fasudil, as highlighted by both NMR and MD simulations. While fasudil has little effect on both aspartate (Figure 2C) and glutamate (Figure S6C) side-chain NMR signals of the protein alone, its effect becomes noticeable when the ligand is added to a solution of the protein already exposed to Ca^2+^ ions (Figure 2E and Figure S6D). In particular, MD simulations show that upon binding to Y125, fasudil is capable of significantly reducing Ca^2+^ interaction with E123, a result that seems to be observed also through NMR experiments where the peak position of E123 in the presence of Ca^2+^ and fasudil moves toward the one of the free form with respect to E123 peak position in the presence of Ca^2+^ alone (Figure S6D). However, the large number of possible contacts enabled by the highly flexible protein conformations allows the interaction with both fasudil and Ca^2+^ to be maintained.

In summary, the integrative use of NMR spectroscopy and MD simulations provided molecular-level insights into the binding mechanisms of α-syn with fasudil in the presence of calcium ions otherwise difficult to obtain with other techniques. By shifting the focus to the amino acid side chains, the primary mediators of interactions with both molecular partners and small molecules, including metal ions, distinct driving forces underlying ligand (i.e., fasudil and Ca^2+^) interactions were elucidated. Furthermore, the data also revealed the capacity of IDP states to interact with more than one ligand simultaneously through similar regions of the protein as revealed from experimental data (i.e., C-terminal region). In the present case the data reveal that α-syn remains competent to engage fasudil even under conditions of elevated Ca^2+^ concentrations, which are known to influence its conformational ensemble and aggregation propensity *in vivo*. This observation suggests that the fasudil interaction with α-syn is structurally resilient to Ca^2+^-induced perturbations. The ability of fasudil to retain binding affinity in Ca^2+^-enriched environments highlights its promise as a therapeutically viable ligand, particularly in the context of neurodegenerative diseases such as Parkinson’s disease, where dysregulated calcium homeostasis is a common pathological feature. In light of the present study, it will be interesting to assess the combined effects of fasudil and calcium ions on protein self-aggregation.

## Methods

Isotopically labeled (^15^N and ^13^C–^15^N) α-syn and C-α-syn were expressed and purified as described in detail in the Supporting Information. NMR experiments were acquired at 298 K and at high field NMR instruments using the parameters and experimental setup reported in the Supporting Information. MD simulation parameters are also reported in the Supporting Information.

## Supplementary Material



## Notes

Additional experimental details, materials and methods, including protein expression protocols, NMR samples details, description of NMR experimental parameters and analysis, and molecular dynamics simulations. The following figures are also available: **S1** - Comparison of calcium binding for α-syn and C-α-syn; **S2** - superimposition of the ^1^H–^15^N spectrum of C-α-syn in the absence and presence of Ca^2+^; **S3** - ^1^H–^15^N spectrum of C-α-syn, with fasudil, and with fasudil and Ca^2+^; **S4** - Chemical shift differences observed upon addition of different ligands for the four nuclei of the backbone; **S5** - ^1^H–^15^N CSP plots obtained upon complex formation as observed by adding the two ligands in different temporal order; **S6** - 2D CACO spectrum and related zooms of C-α-syn with different ligands; **S7** and **S8** - time-resolved interaction map between fasudil and C-α-syn; **S9** - fasudil–tyrosine π–π stacking interactions observed in MD simulations; **S10** - average interaction (%) of fasudil with nearby residues while also interacting with Tyr residues; **S11** - Interaction frequency between Glu and Asp residues of C-α-syn and Ca^2+^; **S12** - C-α-syn-fasudil residence time in the absence and presence of Ca^2+^ (PDF)
