# Supplementary material for: Molecular Interplay of Small Molecules and Calcium Ions with α‑Synuclein Revealed by NMR and Molecular Dynamics Simulations
Source: ACS Chem Neurosci. 2026 Mar 24;17(8):1462–8. doi: 10.1021/acschemneuro.6c00106 (PMC13088183; doi:10.1021/acschemneuro.6c00106)
Supplement: Supplementary file 1 [file cn6c00106_si_001.pdf]

Molecular interplay of small molecules and calcium ions with α-synuclein revealed by NMR and molecular dynamics simulations

Filippo Turchi[a#], Haydar Taylan Turan[b#], Marco Schiavina[a], Giuseppe Brancato[b], Isabella C. Felli[a*], Roberta Pierattelli[a*]*

[a] Department of Chemistry "Ugo Schiff" and Magnetic Resonance Center (CERM), University of Florence, Via L. Sacconi 6, 50019, Sesto Fiorentino, Italy

[b] Scuola Normale Superiore, Piazza dei Cavalieri 7, 56126, Pisa, Italy

*giuseppe.brancato@sns.it, felli@cerm.unifi.it; roberta.pierattelli@unifi.it

[#] These authors contributed equally

Supporting Information

Materials and methods

Protein Expression

The ^{15}N labelled α-synuclein sample was prepared as previously described [1], lyophilized in water and stored at -20°C.

The C-terminal region (112-140) of α-synuclein (C-α-syn) was expressed using the construct MBP-HisTag-3C-αSyn112-140 in *E.coli* strain BL21 (DE3) gold. A colony from a freshly transformed plate was inoculated into 100 mL Luria Bertani (LB) medium and grown at 37 °C until an optical density (OD_{600}) of 0.8-1 was reached. The culture was then transferred in 1 L of LB medium and grown to OD_{600} of 0.7-0.9. To prepare the uniformly ^{15}N-labeled and ^{13}C,^{15}N-labeled peptide, the culture was resuspended in labelled minimal medium supplemented with 1 g/L $^{15}NH_4Cl$ only (Cambridge Isotope Laboratories) and 3 g/L glucose (Sigma-Aldrich) or 1 g/L $^{15}NH_4Cl$ and 3 g/L $^{13}C_6$-d-glucose (Eurisotop). After 1 hour of unlabelled metabolite clearance, it was induced with 1 mM isopropyl-beta-thiogalactopyranoside (IPTG) at 37°C for 16 hours. Cells were harvested by centrifugation, and the pellet was resuspended in 50 mM 2-amino-2-(hydroxymethyl)-1,3-propanediol (TRIS), 150 mM sodium chloride (NaCl) and protease inhibitor cocktail (SIGMA) at pH 8.0. Cells were disrupted by sonication, and the lysate was centrifuged at 20,000 × *g* for 30 min at 4 °C. The soluble fraction was loaded on a HiTrap FF 5 mL column and washed with 5 CV of 20 mM TRIS, 150 mM NaCl and 10 mM imidazole at pH 8. Then, the 3C protease with His_6-tag was loaded in the column with 20 mM TRIS, 150 mM NaCl at pH 8 where the His_6-MBP-tag was cleaved overnight at 4°C. The peptide was eluted in the same buffer, and the His_6-MBP-tag was removed with 20 mM TRIS, 150mM NaCl and 500mM imidazole at pH 8. Then, lyophilized in water and stocked at -20°C.

NMR samples

For the NMR titration of ^{15}N α-synuclein with Ca^{2+}, experiments were acquired using a 5 mm NMR tube containing 550 µL of 0.1 mM α-synuclein in 50 mM TRIS buffer at pH 7.4, with 5% D_2O added for the lock. A stock solution of $CaCl_2 \cdot 4H_2O$ solution was prepared at a final concentration of 5 M in the same buffer. Aliquots of the $CaCl_2$ stock solution were added to reach the following molar equivalents of α-synuclein to calcium ions: 1:0, 1:0.5, 1:1, 1:2, 1:4, 1:8, 1:12, 1:16, 1:24, 1:48, 1:60, 1:90, 1:120, 1:180, 1:240.

For the NMR titration of ^{15}N C-α-syn with Ca^{2+}, experiments were acquired using a 5 mm NMR tube containing 550 μL of 0.16 mM protein in 25 mM TRIS buffer at pH 7.4, with 5% D_2O added for the lock. A stock solution of $CaCl_2 \cdot 4H_2O$ solution was prepared at a final concentration of 5 M in the same NMR buffer. Aliquots of the $CaCl_2$ stock solution were added to reach the following molar equivalents of C-term to calcium ions: 1:0, 1:2, 1:4,1:6, 1:8, 1:16, 1:32, 1:64, 1:128, 1:256.

For the NMR titrations of ^{13}C,^{15}N C-α-syn with fasudil [5-(1,4-diazepane-1-sulfonyl) isoquinoline] and Ca^{2+}, experiments were acquired in a 5 mm NMR tube containing 500 μL of 0.2 mM protein in 25 mM TRIS buffer with the addition of 13.8 μM of DSS (sodium 2,2-dimethyl-2-silapentane-5-sulfonate), pH 7.4 and 5% D_2O was added for the lock. A stock solution of $CaCl_2 \cdot 4H_2O$ and another stock solution of fasudil were prepared with a final concentration of 25 mM in the same NMR buffer.

An NMR titration was performed by adding fasudil to the ^{13}C,^{15}N C-α-syn solution, followed by the addition of Ca^{2+}. A second titration was carried out by adding Ca^{2+} first, followed by the addition of fasudil. In both titrations, the following molar ratios of (C-α-syn):(first ligand) were used: 1:0, 1:1, 1:2, 1:4, 1:8, and 1:16. Subsequently, the second ligand was added to obtain the following ratios of (C-α-syn):(first ligand):(second ligand): 1:16:1, 1:16:2, 1:16:4, 1:16:8, and 1:16:16.

DSS was used as an internal standard to determine chemical shift values.

NMR spectroscopy

The following spectrometers were used:

- Bruker AVANCE III spectrometer operating at 950.20 MHz ^{1}H, 238.93 MHz ^{13}C, and 96.28 MHz ^{15}N frequencies, equipped with a cryogenically cooled probe head optimized for ^{1}H-direct detection (TCI). Namely, *950.*

- Bruker AVANCE NEO spectrometer operating at 899.64 MHz ^{1}H, 226.21 MHz ^{13}C, and 91.16 MHz ^{15}N frequencies equipped with a cryogenically cooled probe head optimized for ^{1}H-direct detection (TCI). Namely, *900.*

- Bruker AVANCE NEO spectrometer operating at 700.06 MHz ^{1}H, 176.03 MHz ^{13}C, and 70.94 MHz ^{15}N frequencies equipped with a cryogenically cooled probe head optimized for ^{13}C-direct detection (TXO). Namely, *700.*

To follow the interaction of ^{15}N α-synuclein with Ca^{2+} a series of 2D HN HSQC [2] experiments were recorded at *950*. The carrier frequency for ^{1}H was set at 4.7 ppm; for the ^{15}N, the carrier was set at 120 ppm. Hard pulses were used both for ^{1}H and ^{15}N excitation. A recycle delay of 1.1 s was used. Solvent suppression was achieved through the 3:9:19 pulse scheme [3].

To follow the interaction of ^{15}N C-α-syn with Ca^{2+} and fasudil a series of 2D HN BEST-TROSY [4] experiments were recorded at *900*. The carrier frequency for ^{1}H was set at 4.7 ppm; for the ^{15}N, the carrier was set at 121 ppm. Pc9 [5] and Eburp shaped pulses [6] of durations of 1781 and 1344 μs, respectively, were used for $^{1}H^{N}$ band-selective $\pi/2$ flip angle rotation. Reburp shaped [6] pulses of durations of 1135 μs were used for $^{1}H^{N}$ band-selective π flip angle rotation. Bip [7] and Reburp shaped pulses of durations of 500 and 1067 μs, were used for $^{15}N^{H}$ band-selective $\pi/2$ and π flip angle rotations respectively. A recycle delay of 0.34 s was used.

To follow the interaction of ^{13}C, ^{15}N C-α-syn with calcium ions and fasudil a series of 2D CACO [1,8] and 2D HC-TROSY [9] experiments were recorded at *700*. For the CACO the ^{13}C pulses were cantered at 176.2 ppm and 48.7 ppm for C′ and C^{α} respectively. For the HC-TROSY the $^{13}C^{aro}$ pulses were cantered at 125 ppm. The carrier frequency for ^{15}N was set at 123 ppm. The ^{1}H carrier was set at 4.7 ppm. For each increment of the 2D CACO experiment, the in-phase (IP) and antiphase (AP) components were acquired and properly combined to achieve IPAP virtual decoupling [10].

To properly assign the resonances of tyrosine side chains the HDCB and HECB experiments were also used [11]. Here, the ^{13}C pulses were set at 125 ppm and 38 ppm for C^{aro} and C^{β}, respectively the ^{1}H carrier was set at 4.7 ppm.

For the CACO Q5 and Q3 shaped [12] pulses of durations of 300 and 231 μs, respectively, were used for ^{13}C band-selective $\pi/2$ and π flip angle pulses, except for the adiabatic π pulse to invert both C′ and C^{α} (smoothed chirp 500 μs, 20% smoothing, 80 kHz sweep width, 11.3 kHz radio frequency field strength). In the HDCB and HECB experiments, the same shaped pulses were used. Here a more selective pulse was also exploited to irradiate only the C^{β} spins (Q3-shaped π pulse of duration of 900 μs).

The sequence specific assignment of C-α-syn was obtained for comparison with the available one of α-synuclein and confirmed by using a series of standard 2D and 3D NMR experiments (CON[8], hcaCON^{pro} [13], hCACO [14], hCBCACO [8], hCCCO [14] and hCBCACON[13]) acquired at *900* employing

standard parameters used for biomolecular NMR experiments. Carbon-13 homonuclear decoupling was achieved through the IPAP virtual decoupling approach [10].

The assignment of the side chain of tyrosine residues was obtained by combining the information from the HC-TROSY, the HBCD and HBCE experiments. An HC TROSY experiment tailored for the aromatic residues [9] exploiting the WATERGATE approach [3] was acquired at *700*. Specifically, we acquired a modified version in which the constant time evolution period was increased, from 8.8 ms to 17.6 ms, to achieve higher resolution in the indirect dimension enabling the detection of well resolved cross peaks.

CSP calculation

To measure the backbone chemical shift perturbation (CSP) in ^{1}H-^{15}N spectra the following equation was employed:

$$CSP = \sqrt{\delta_H{}^2 + (k * \delta_N)^2}$$

where δ_H and δ_N are the differences in the chemical shift value between the peak position in a titration point and the initial chemical shift value for the same peak in the proton and nitrogen dimensions, respectively, and k a constant; for this work we used k = 0.1 [15].

To determine the backbone CSP in the CACO spectra the following formula was instead employed:

$$CSP = \sqrt{\delta_{C'}{}^2 + \delta_{C^\alpha}{}^2}$$

The CSP values of only the well resolved peaks of C-α-syn were used to monitor the interaction with calcium ions and fasudil.

Molecular Dynamics Simulations

All molecular dynamics (MD) simulations were performed using the Amber FF99SB-disp force field [16,17] in Amber 20 [18]. The equations of motion were integrated with a time step of 2 fs, and all bonds involving hydrogen atoms were constrained using the SHAKE algorithm[19]. The system, comprising the Ile112–Ala140 segment of C-α-syn, was solvated in a truncated octahedral box of TIP4PD-disp [20] water with a 14.0 Å buffer around the solute.

To achieve a 1:5 α-synuclein: Ca^{2+} ratio, five Ca^{2+} cations were added, corresponding to a 30 mM ion concentration. The latter ratio falls within the range of concentrations tested experimentally during the NMR titration experiments (from 1:2 to 1:256), and it allowed us to reproduce well the calcium modulation effect observed experimentally on protein/fasudil interaction.

Molecular docking of fasudil into three different tyrosine residues of C-α-syn was performed using AutoDock Vina [21]. The protein structure was prepared by optimizing protonation states, adding missing hydrogen atoms, and assigning Gasteiger charges [22] using AutoDockTools (ADT) [23]. The fasudil ligand was prepared by optimizing its geometry and protonation state for docking. A docking grid was centered on each tyrosine residue with a box size of 20 × 20 × 20 Å to allow sufficient sampling of binding poses. Docking was performed with an exhaustiveness of 8, generating 10 binding modes ranked by Vina's scoring function [24]. The binding poses were analyzed based on binding affinity and key interactions (hydrogen bonding, hydrophobic contacts, and π-stacking), and the most stable and energetically favorable conformations were selected for subsequent MD simulations. This approach resulted in three distinct C-α-syn-fasudil complexes, which served as the starting structures for MD simulations.

The C-α-syn-fasudil complexes were positioned at the center of the simulation box, followed by energy minimization, heating to 300 K, and equilibration for 2 ns in the *NVT* ensemble. Production MD simulations were carried out according to the *NpT* ensemble for 2 μs. A total of six simulations were performed: three with both $Ca^{2+}$and fasudil, and three with only fasudil along with C-α-syn. Results from all three simulations of each set were merged for analysis.

C-α-syn-fasudil residence time distributions in the absence and presence of calcium ions were evaluated from MD simulations considering a cut-off distance of 5 Å among any atom of the two chemical species, while fasudil's dissociation constants were estimated following the same approach of ref. 16.

Supplementary Figures

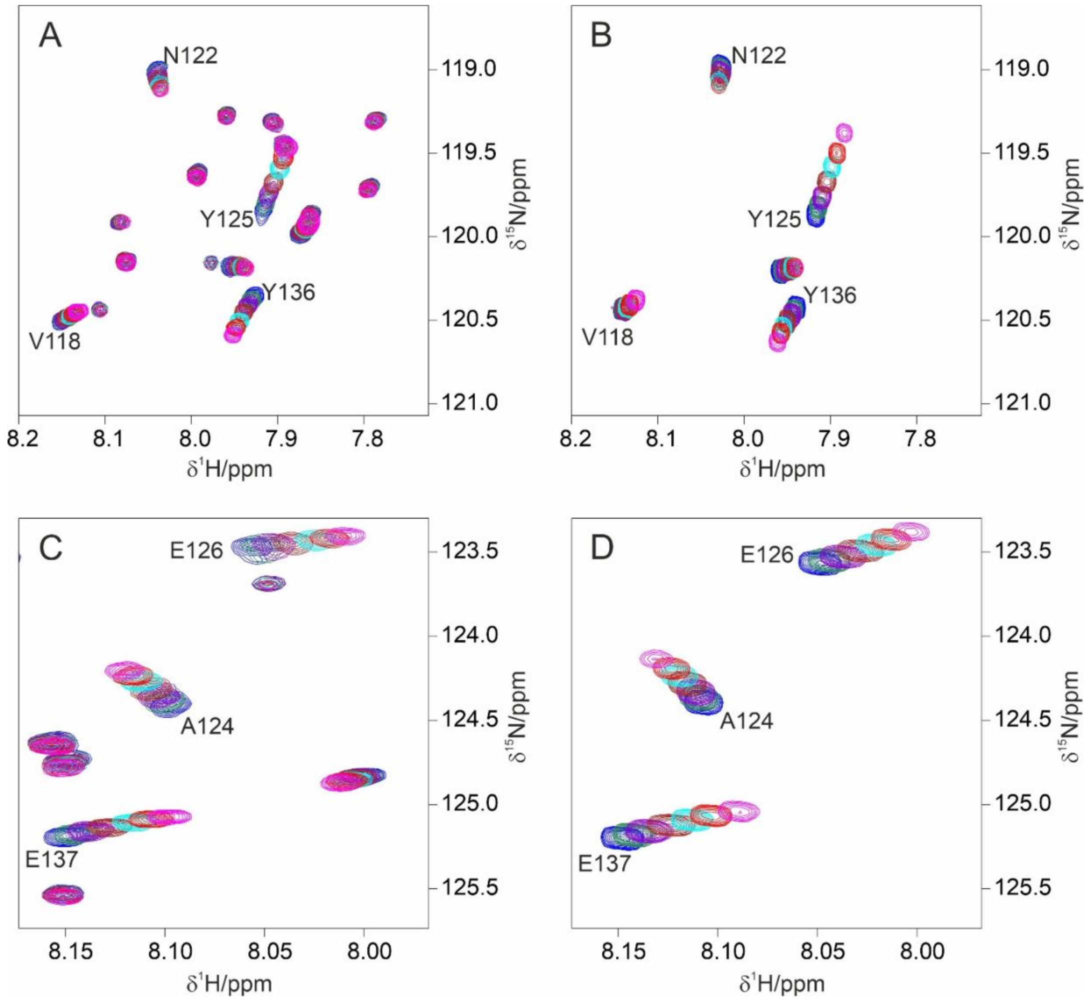

Figure S1. Two key regions of the 2D HN spectra showing the overlay of α-synuclein (A and C) and C-α-syn (B and D) upon the addition of calcium ions. The spectra of the free form of α-syn and upon addition of 0.4, 1.2, 2.4, 4.8, 9.0 and 18.0 mM of Ca^{2+} are reported in blue, green, dark violet, burgundy, cyan, red and light violet contours in panels A and C. The spectra of the free form of C-α-syn and upon addition of 0.64, 1.3, 2.6, 5.1, 10.2 and 20.5 mM of Ca^{2+} are reported in blue, green, dark violet, burgundy, cyan, red and light violet contours in panels B and D. In the case of α-synuclein and C-α-syn the protein concentration was 0.1 mM and 0.16 mM, respectively.

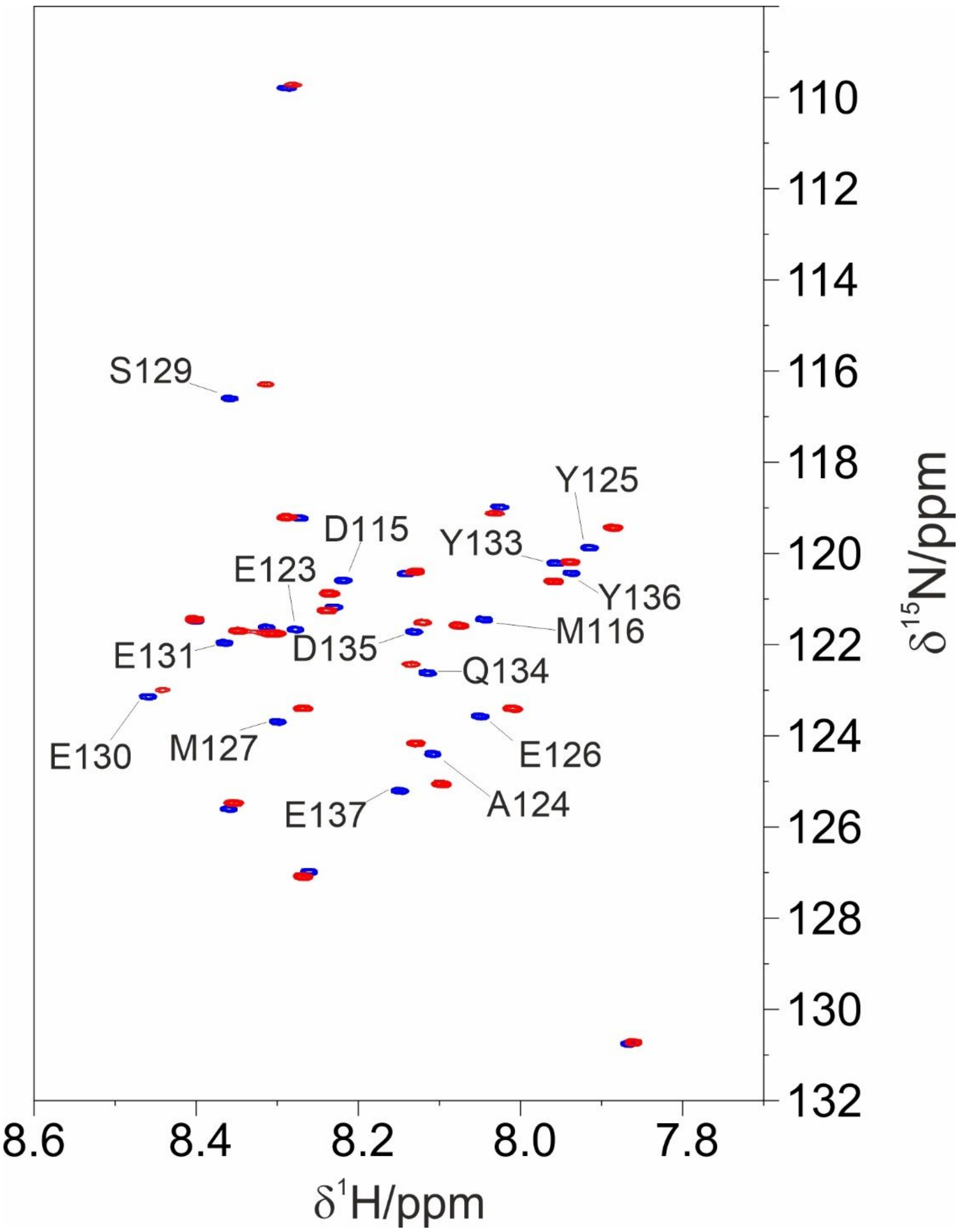

Figure S2. The figure shows the superimposition of ^{1}H-^{15}N spectrum of ^{15}N C-α-syn (blue) with the one obtained upon addition of 20.5 mM of Ca^{2+} (red). The C-α-syn concentration was 0.16 mM.

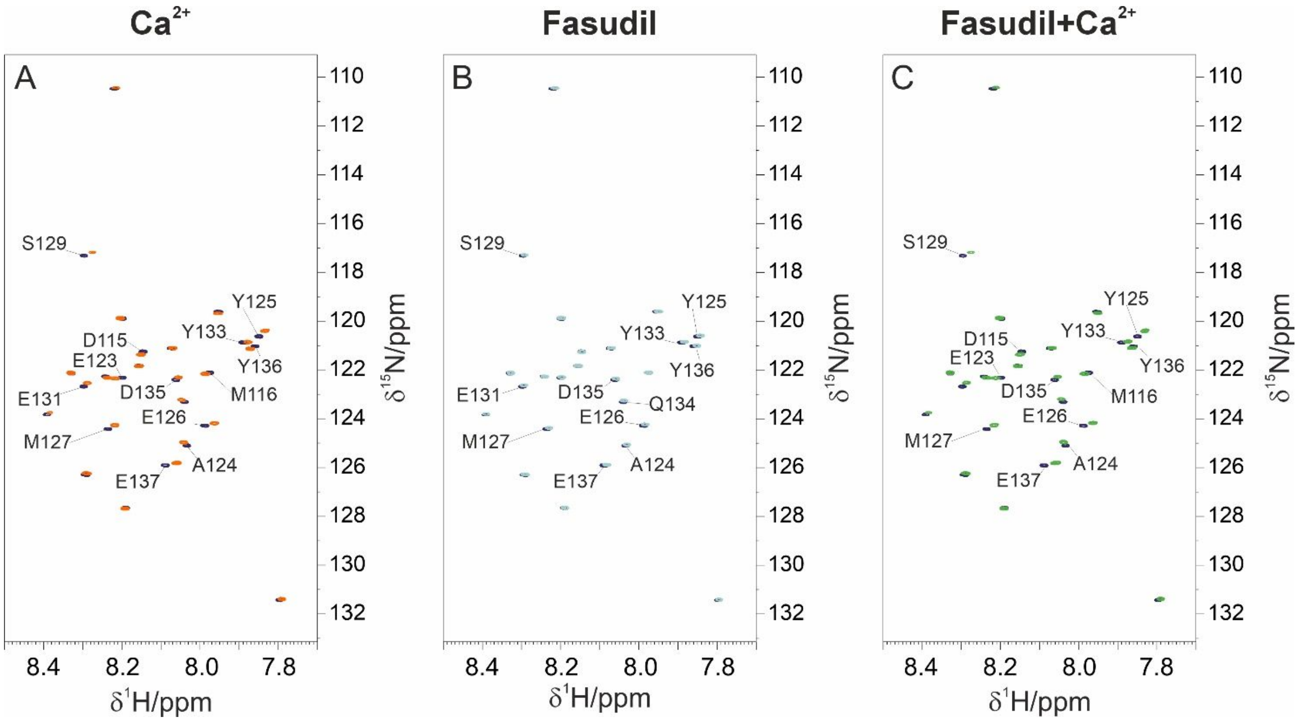

Figure S3. Panels A and B display the superimposition of the reference ^{1}H-^{15}N HSQC spectrum of ^{15}N C-α-syn (0.2 mM) in blue with the spectra recorded after the addition of Ca^{2+} (panel A, orange), or fasudil (panel B, light blue) in molar ratios of 16:1 respect to C-α-syn (3.2 mM ligand:0.2 mM protein). Panel C shows the spectra obtained at 16:0:1, and 16:16:1 fasudil:Ca^{2+}:protein molar ratios (3.2 mM fasudil:0 µM Ca^{2+}:0.2 mM protein and 3.2 mM fasudil:3.2 mM Ca^{2+}:0.2 mM protein) in light blue and light green, respectively.

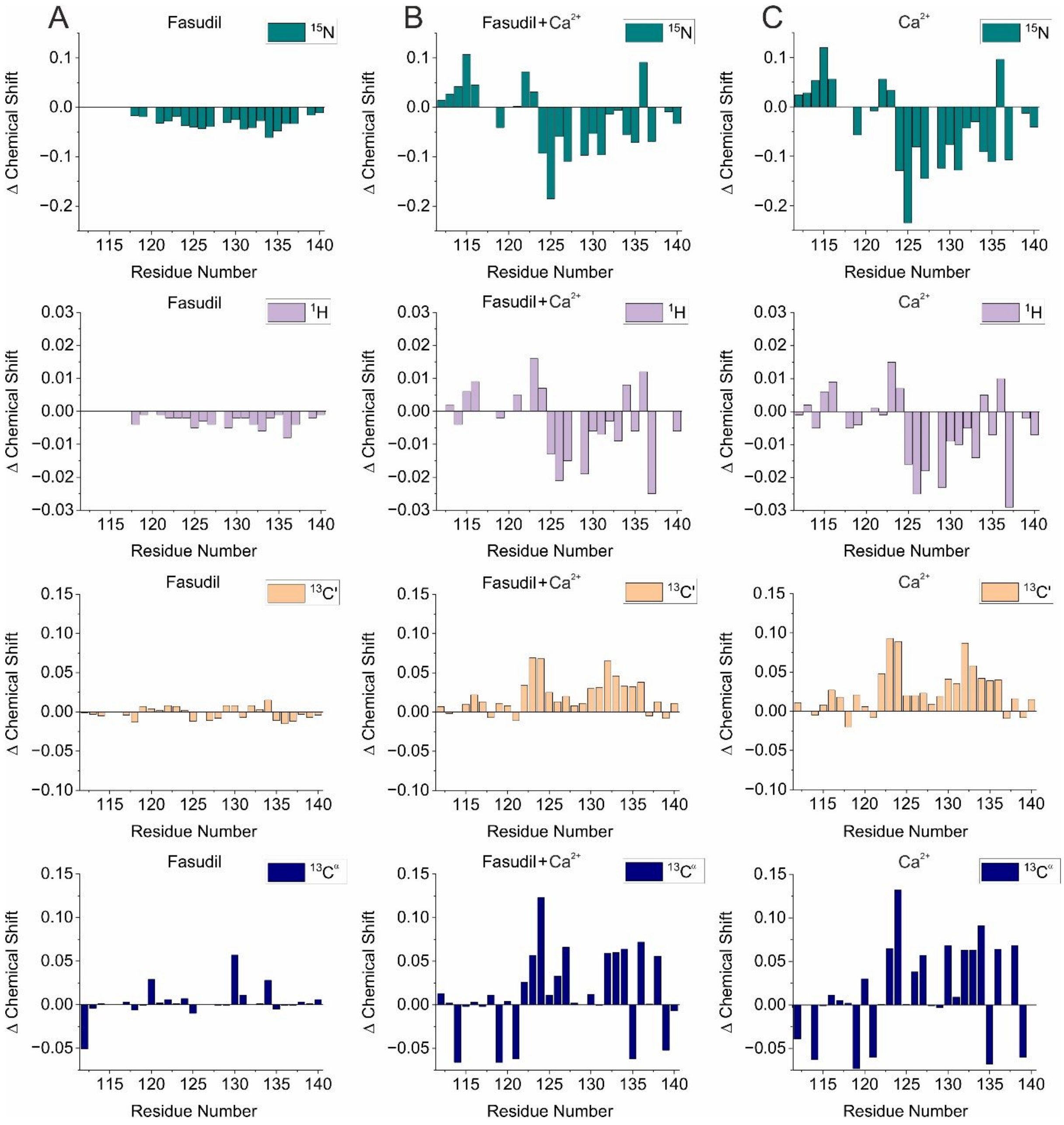

Figure S4. Chemical shift differences observed upon addition of different ligands for the four nuclei of the backbone ($^{1}H^{N}$, ^{15}N, $^{13}C'$ and $^{13}C^{\alpha}$ reported from the top to the bottom row). Column A shows the plots obtained upon the addition of 3.2 mM fasudil to 0.2 mM C-α-syn. Column B shows the plots obtained upon the addition of 3.2 mM fasudil and 3.2 mM Ca^{2+} to 0.2 mM C-α-syn. Column C shows the plots obtained upon the addition of 3.2 mM of calcium ions to 0.2 mM C-α-syn.

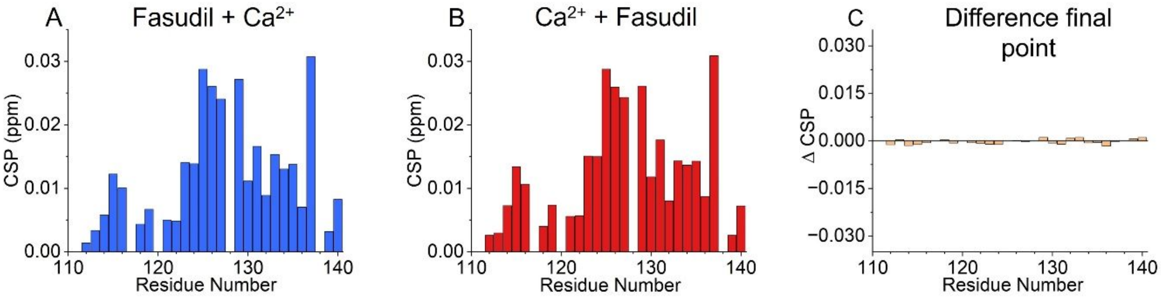

Figure S5. Panel A shows the ^{1}H–^{15}N CSP plot comparing C-α-syn in its free state and after addition 3.2 mM fasudil and 3.2 mM Ca^{2+} to 0.2 mM C-α-syn. Panel B shows the corresponding plot obtained after addition of 3.2 mM Ca^{2+} and 3.2 mM fasudil to 0.2 mM C-α-syn. Panel C displays the difference between the two plots.

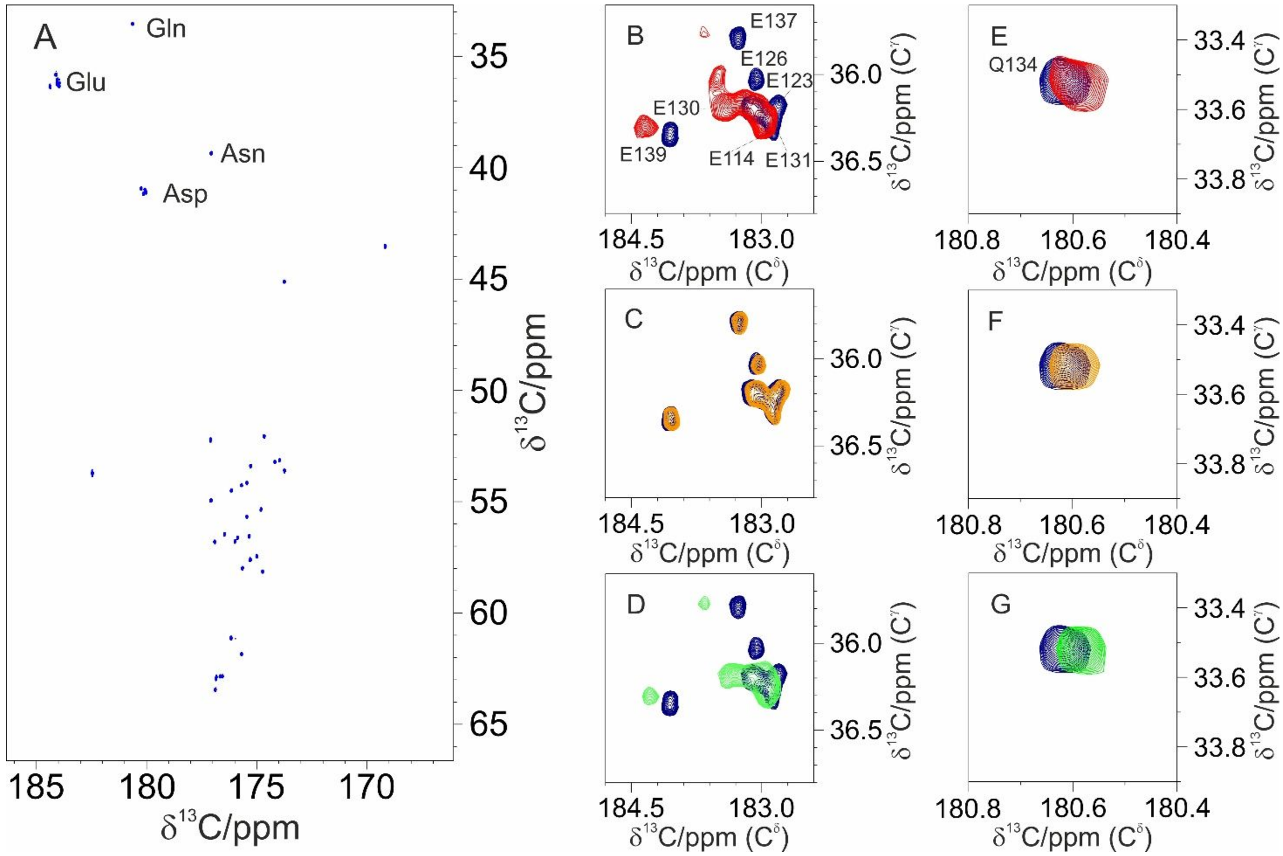

Figure S6. Panel A shows the 2D CACO spectrum of ^{13}C-^{15}N C-α-syn, at the concentration of 0.2 mM. The spectra reported in blue correspond to the free state of the protein. Panels B, C, and D display zoomed-in regions corresponding to the resonances arising from glutamate residues (C^{γ}-C^{δ}) while panels E, F, and G show zoomed-in regions highlighting the resonances of glutamine residues (C^{γ}-C^{δ}). Panels B and E show in red the spectra after the addition of 3.2 mM Ca^{2+}; panels C and F show in orange the spectra obtained upon the addition of 3.2 mM fasudil; and panels D and G show in green the spectra after the addition of 3.2 mM Ca^{2+} and 3.2 mM fasudil.

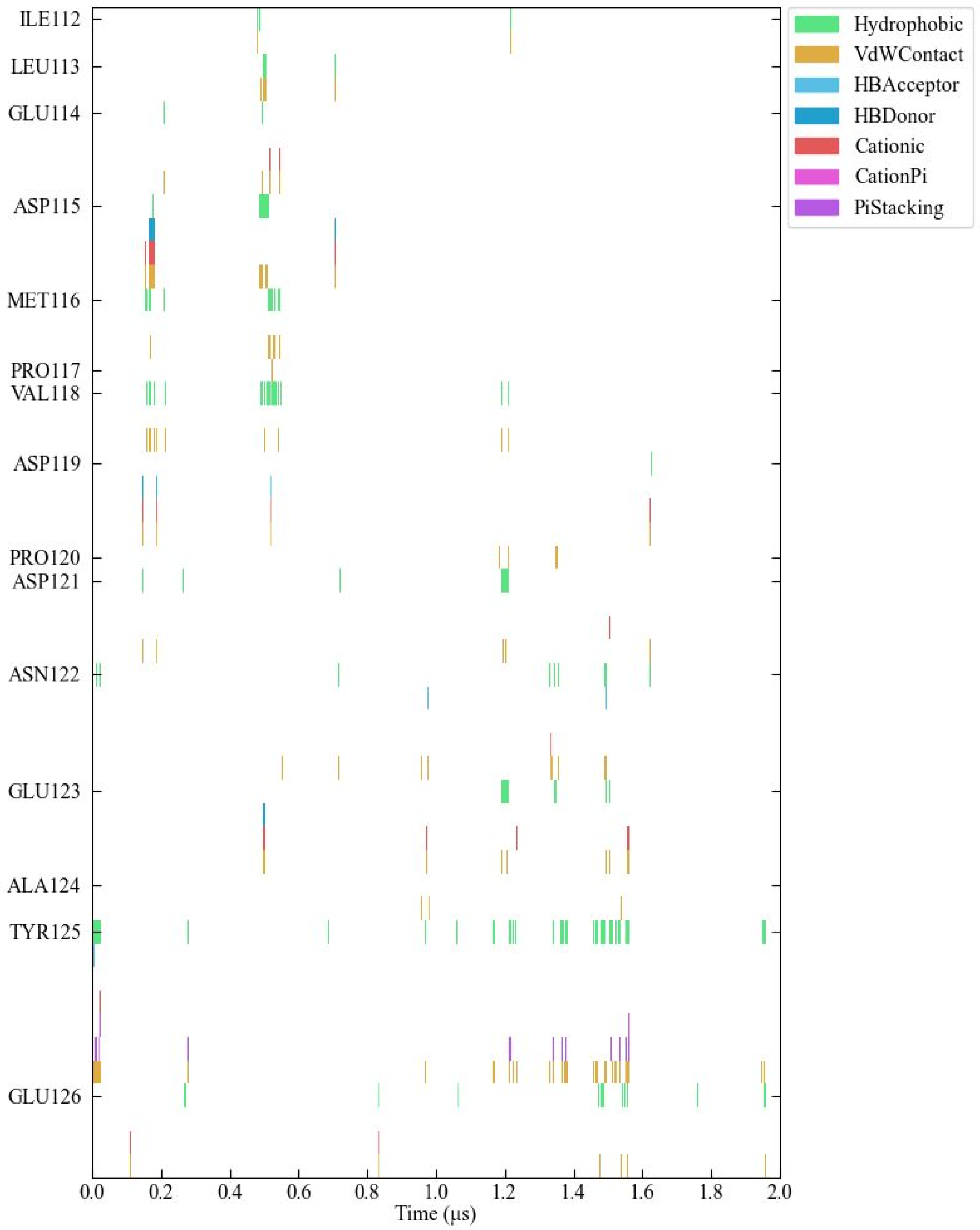

Figure S7. Time-resolved interaction map between fasudil (residues from 112 to 126) and C-α-syn in the absence of Ca^{2+} ions. Each horizontal line corresponds to a specific interaction type (e.g., hydrogen bond, π–stacking), as indicated in the plot legend, and shows when the interaction occurs throughout the simulation. Each line segment represents 100 ps of simulation time.

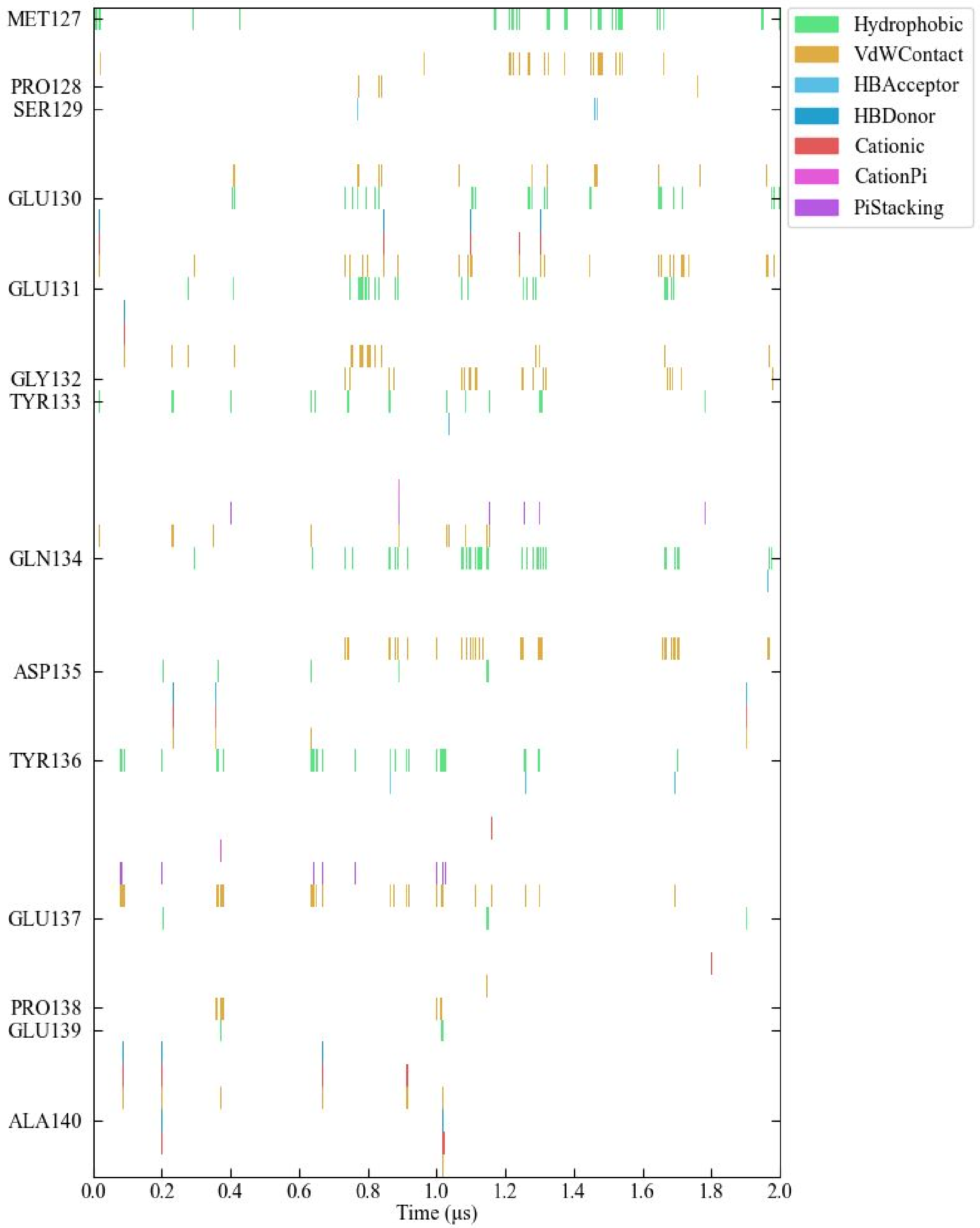

Figure S8. Time-resolved interaction map between fasudil (residues from 127 to 140) and C-α-syn in the absence of Ca^{2+} ions. Each horizontal line corresponds to a specific interaction type (e.g., hydrogen bond, π–stacking), as indicated in the plot legend, and shows when the interaction occurs throughout the simulation. Each line segment represents 100 ps of simulation time.

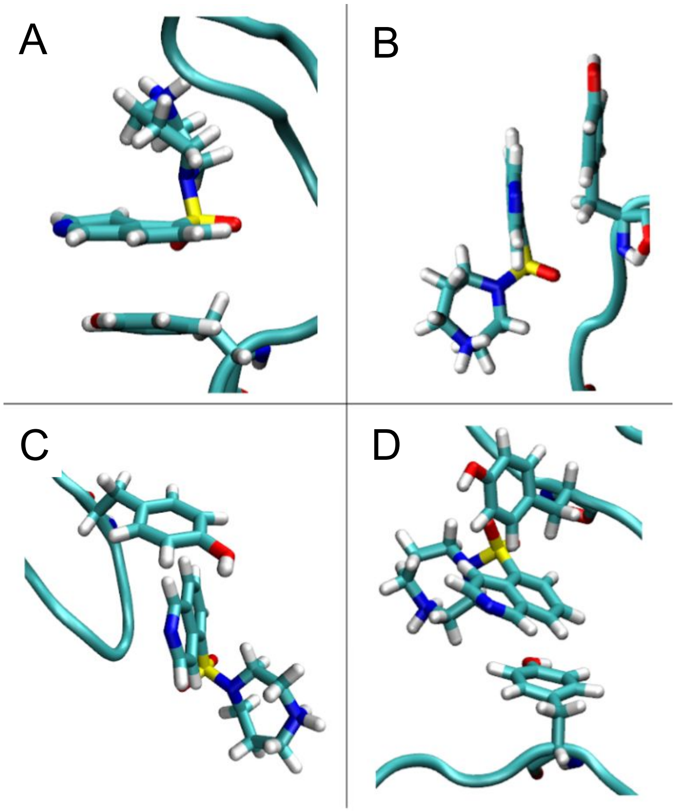

Figure S9. Types of fasudil–tyrosine π–π stacking interactions observed in MD simulations: (A) sandwich, (B) parallel-displaced, (C) T-shaped, and (D) a combination of multiple interaction types.

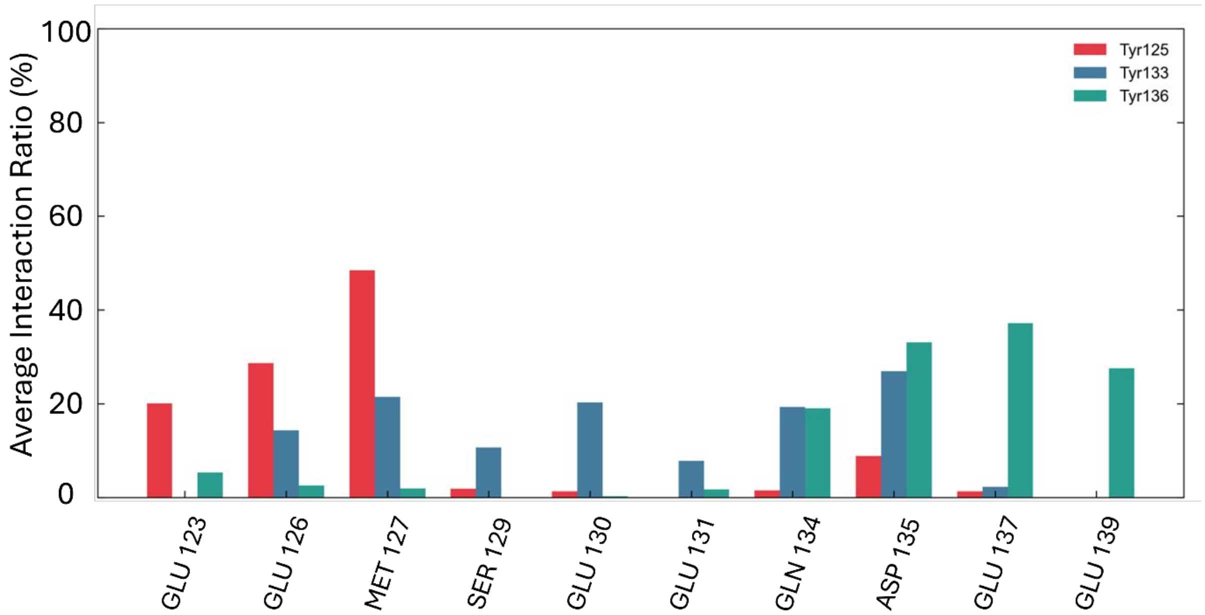

Figure S10: Average interaction (%) of fasudil with nearby residues while also interacting with Tyr125 (red), Tyr133 (blue), and Tyr136 (green) as issuing from independent MD simulations in absence of Ca^{2+} (only configurations displaying fasudil-Tyr interaction were retained in the corresponding analysis). Results highlight the correlations between Tyr's residues and a few other residues during C-α-syn-fasudil interaction.

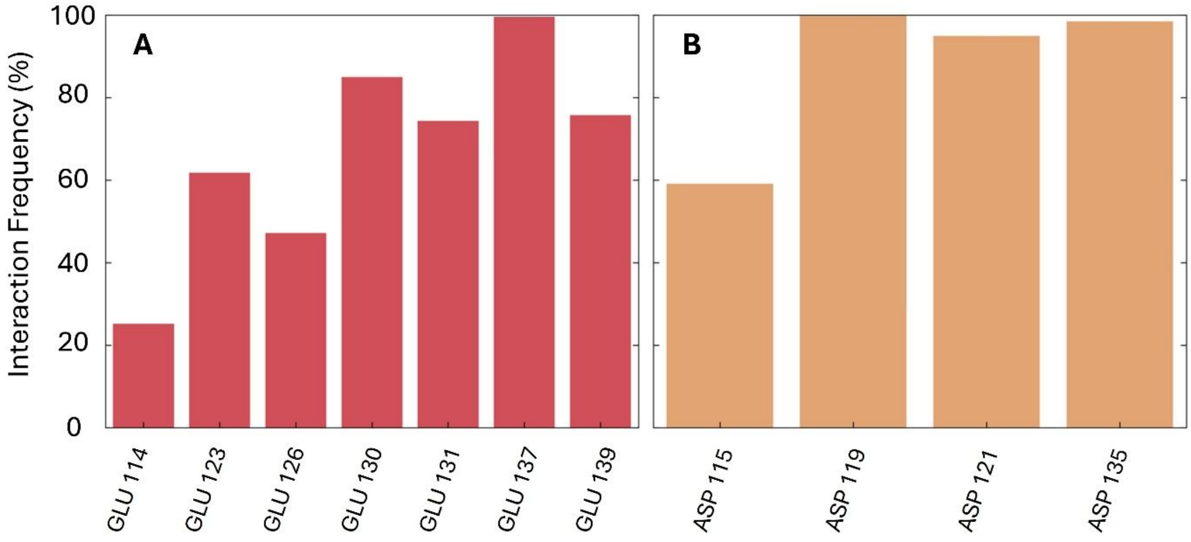

Figure S11: Interaction frequency (%) between Glu (red, panel A) and Asp (orange, panel B) residues of C-α-syn and Ca^{2+} as issuing from MD simulations.

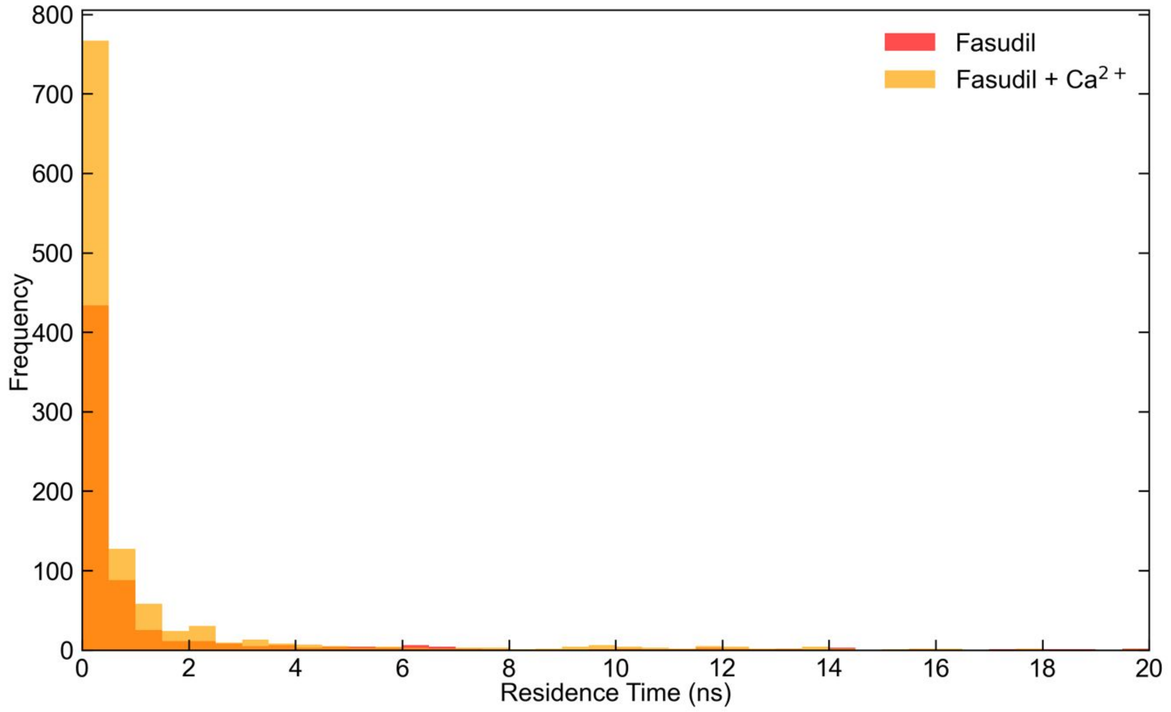

Figure S12: C-α-syn-fasudil residence time distribution from MD simulations, in absence (dark orange) and presence of Ca^{2+} (light orange): the computed average residence time is 2.8 ns and 2.1 ns, respectively, with a noticeable time reduction that parallels well the observed decrease of interaction probability (see Figure 3).

References

(1) Pontoriero, L.; Schiavina, M.; Murrali, M. G.; Pierattelli, R.; Felli, I. C. Monitoring the interaction of α-synuclein with calcium ions through exclusively heteronuclear nuclear magnetic resonance experiments. *Angew. Chem.* 2020, *59* (42), 18537–18545. https://doi.org/10.1002/anie.202008079.

(2) Mori, S.; Abeygunawardana, C.; Johnson, M. O.; Vanzijl, P. C. M. Improved sensitivity of HSQC spectra of exchanging protons at short interscan delays using a new Fast HSQC (FHSQC) detection scheme that avoids water saturation. *J. Magn. Reson. B* 1995, *108* (1), 94–98. https://doi.org/10.1006/jmrb.1995.1109.

(3) Sklenar, V.; Piotto, M.; Leppik, R.; Saudek, V. Gradient-tailored water suppression for ^{1}H-^{15}N HSQC experiments optimized to retain full sensitivity. *J. Magn. Reson. A* 1993, *102* (2), 241–245. https://doi.org/10.1006/jmra.1993.1098.

(4) Solyom, Z.; Schwarten, M.; Geist, L.; Konrat, R.; Willbold, D.; Brutscher, B. BEST-TROSY experiments for time-efficient sequential resonance assignment of large disordered proteins. *J Biomol NMR* 2013, *55*, 311–321. https://doi.org/10.1007/s10858-013-9715-0.

(5) Kupce, E.; Freeman, R. Polychromatic selective pulses. *J. Magn. Reson. A* 1993, *102* (1), 122–126. https://doi.org/10.1006/jmra.1993.1079.

(6) Geen, H.; Freeman, R. Band-selective radiofrequency pulses. *J. Magn. Reson. (1969)* 1991, *93* (1), 93–141. https://doi.org/10.1016/0022-2364(91)90034-Q.

(7) Smith, M. A.; Hu, H.; Shaka, A. J. Improved broadband inversion performance for NMR in liquids. *J. Magn. Reson.* 2001, *151*, 269–283. https://doi.org/10.1006/jmre.2001.2364.

(8) Bermel, W.; Bertini, I.; Duma, L.; Felli, I. C.; Emsley, L.; Pierattelli, R.; Vasos, P. R. Complete assignment of heteronuclear protein resonances by protonless NMR spectroscopy *Angew. Chem.* 2005, *44* (20), 3089–3092. https://doi.org/10.1002/anie.200461794.

(9) Pervushin, K.; Riek, R.; Wider, G.; Wu, K. Transverse relaxation-optimized spectroscopy (TROSY) for NMR studies of aromatic spin systems in ^{13}C-labeled proteins. *J. Am. Chem. Soc.* 1998, *120*, 6394–6400.

(10) Felli, I. C.; Pierattelli, R. Spin-state-selective methods in solution- and solid-state biomolecular ^{13}C NMR. *Prog Nucl Magn Reson Spectrosc* 2015, *84–85*, 1–13. https://doi.org/10.1016/j.pnmrs.2014.10.001.

(11) Prompers, J. J.; Groenewegen, A.; Hilbers, C. W.; Pepermans, H. A. M. Two-Dimensional NMR experiments for the assignment of aromatic side chains in ^{13}C-labeled proteins. *J. Magn. Reson.* 1998, *130*, 68–75.

(12) Emsley, L.; Bodenhausen, G. Optimization of shaped selective pulses for NMR using a quaternion description of their overall propagators. *J. Magn. Reson. (1969)* 1992, *97* (1), 135–148. https://doi.org/10.1016/0022-2364(92)90242-Y.

(13) Bermel, W.; Bertini, I.; Felli, I. C.; Pierattelli, R. Speeding up ^{13}C direct detection biomolecular NMR spectroscopy. *J. Am. Chem. Soc.* 2009, *131*, 15339–15345. https://doi.org/10.1021/ja9058525.

(14) Bermel, W.; Bertini, I.; Csizmok, V.; Felli, I. C.; Pierattelli, R.; Tompa, P. H-start for exclusively heteronuclear NMR spectroscopy: The case of intrinsically disordered proteins. *J. Magn. Reson.* 2009, *198* (2), 275–281. https://doi.org/10.1016/j.jmr.2009.02.012.

(15) Williamson, M. P. Using chemical shift perturbation to characterise ligand binding. *Prog. Nucl. Magn. Reson. Spectrosc.* 2013, *73*, 1–16. https://doi.org/10.1016/j.pnmrs.2013.02.001.

(16) Piana, S.; Robustelli, P.; Tan, D.; Chen, S.; Shaw, D. E. Development of a force field for the simulation of single-chain proteins and protein-protein complexes. *J. Chem. Theory Comput.* 2020, *16* (4), 2494–2507. https://doi.org/10.1021/acs.jctc.9b00251.

(17) Robustelli, P.; Piana, S.; Shaw, D. E. Developing a molecular dynamics force field for both folded and disordered protein states. *Proc. Natl. Acad. Sci. U.S.A.* 2018, *115* (21), E4758–E4766. https://doi.org/10.1073/pnas.1800690115.

(18) D.A. Case; K. Belfon; I.Y. Ben-Shalom; S.R. Brozell; D.S. Cerutti; T.E. Cheatham; III V.W.D. Cruzeiro; T.A. Darden; R.E. Duke; G. Giambasu; M.K. Gilson; H. Gohlke; A.W. Goetz; R Harris; S. Izadi; S.A. Iz-; mailov; K. Kasavajhala; A. Kovalenko; R. Krasny; T. Kurtzman; T.S. Lee; S. LeGrand; P. Li; C. Lin; J. Liu; T. Luchko; R. Luo; V. Man; K.M. Merz; Y. Miao; O. Mikhailovskii; G. Monard; H. Nguyen; A. Onufriev; F. Pan; S. Pantano; R. Qi; D.R. Roe; A. Roitberg; C. Sagui; S. Schott-Verdugo; J. Shen; C.L. Simmerling; N.R.; Skrynnikov; J. Smith; J. Swails; R.C. Walker; J. Wang; L. Wilson; R.M. Wolf; X. Wu; Y. Xiong; Y. Xue; D.M. York; P.A. Kollman. AMBER. 2020. University of California, San Francisco.

(19) Ryckaert, J.-P.; Ciccotti, G.; Berendsen, H. J. C. Numerical integration of the cartesian equations of motion of a system with constraints: Molecular dynamics of n-alkanes. *J. Comput. Phys.* 1977, *23* (23), 321–341.

(20) Piana, S.; Donchev, A. G.; Robustelli, P.; Shaw, D. E. Water dispersion interactions strongly influence simulated structural properties of disordered protein states. *J. Phys. Chem. B* 2015, *119* (16), 5113–5123. https://doi.org/10.1021/jp508971m.

(21) Trott, O.; Olson, A. J. AutoDock vina: improving the speed and accuracy of docking with a new scoring function, efficient optimization, and multithreading. *J. Comput. Chem.* 2010, *31* (2), 455–461. https://doi.org/10.1002/jcc.21334.

(22) Gasteiger, J.; Marsili, M. A new model for calculating atomic charges in molecules. *Tetrahedron Letters No* 1978, *19* (34), 3181–3184.

(23) Morris, G. M.; Ruth, H.; Lindstrom, W.; Sanner, M. F.; Belew, R. K.; Goodsell, D. S.; Olson, A. J. Software news and updates AutoDock4 and AutoDockTools4: Automated docking with selective receptor flexibility. *J. Comput. Chem.* 2009, *30* (16), 2785–2791. https://doi.org/10.1002/jcc.21256.

(24) Huey, R.; Morris, G. M.; Olson, A. J.; Goodsell, D. S. Software news and update a semiempirical free energy force field with charge-based desolvation. *J. Comput. Chem.* 2007, *28* (6), 1145–1152. https://doi.org/10.1002/jcc.20634.
